# Effects of Suppository Acetaminophen, Bupivacaine Wound Infiltration, and Caudal Block With Bupivacaine on Postoperative Pain in Pediatric Inguinal Herniorrhaphy

**DOI:** 10.5812/aapm.3551

**Published:** 2012-04-01

**Authors:** Seyed Abbas Hosseini Jahromi, Sadegh Sadeghi poor, Seyedeh Masoumeh Hosseini Valami, Amir Javadi

**Affiliations:** 1 Department of Anesthesiology, Qazvin University of Medical Sciences, Shahid Rajaee Hospital, Qazvin, Iran; 2 Department of Surgery, Qazvin University of Medical Sciences, Qazvin, Iran; 3 Department of Social Medicine, Qazvin University of Medical Sciences, Qazvin, Iran

**Keywords:** Bupivacaine, Anesthesia, Caudal, Pediatrics, Analgesia, Suppositories, Acetaminophen

## Abstract

**Background:**

The control of postoperative pain is important in children, and poor pain control leads to organ dysfunction and behavioral problems.

**Objectives:**

We compared the analgesic effects of suppository acetaminophen, bupivacaine wound infiltration, and caudal block with bupivacaine on postoperative pain in pediatric inguinal herniorrhaphy.

**Patients and Methods:**

In this double-blinded, randomized controlled clinical trial, 90 children of American Society of Anesthesiologists (ASA) grade I-II, aged between 3 months and 7 years, and scheduled for elective unilateral inguinal herniorrhaphy under general anesthesia were assigned to three equal groups. Patients in the first group received 20 mg/kg of suppository acetaminophen. In the second group, 2 mg/kg of 0.5% bupivacaine was infiltrated in the incisional site, and in the third group, a caudal block was performed with 0.75 mL/kg of 0.25% bupivacaine. The Face, Legs, Activity, Cry, Consolability (FLACC) pain scale was applied 30 minutes after operation. Thereafter, the FLACC score was obtained every hour during the next 6 hours. If the FLACC score was 4 or over, we administered 0.5 mg/kg of intravenous meperidine. The data was transferred to SPSS-10 software and analyzed statistically with chi-square and analysis of variance tests. *P* < 0.05 was considered significant.

**Results:**

The mean analgesic duration in the acetaminophen, bupivacaine infiltration, and caudal block groups was 4.07, 5.40, and 5.37 hours, respectively. Significant differences were not observed between the bupivacaine infiltration and caudal block groups (*P* = 0.9), but the differences between the bupivacaine infiltration and acetaminophen groups (*P* = 0.034) and the caudal block and acetaminophen groups (*P* = 0.039) were significant. With regard to meperidine administration, significant differences were not observed between the bupivacaine infiltration and caudal block groups (*P* = 0.848), but significant differences were observed between these two groups and the acetaminophen group (*P* < 0.05).

**Conclusions:**

Patients in the bupivacaine infiltration and caudal block groups had less postoperative pain than those in the acetaminophen group and received lower amount of meperidine. We concluded that in children, bupivacaine infiltration and caudal block with bupivacaine produce better analgesia than suppository acetaminophen. It seems that bupivacaine infiltration is better than caudal block because of its simplicity, lower incidence of complications, and failure rate.

## 1. Background

Individual variations in the response to pain are influenced by the genetic makeup, cultural background, age, and gender. Certain patient populations are at risk of inadequate pain control and require special attention, including pediatric patients, geriatric patients, and patients with difficulty in communication (1).

Pain may trigger biochemical and physiologic stress responses and leads to impairments in pulmonary, cardiovascular, neuroendocrinal, gastrointestinal, immunological, and metabolic function even in children and newborns (2, 3). Effective pain therapies to block or modify the physiologic responses to pain and stress have become an essential component of modern pediatric anesthesia and surgical practice (2).

In pediatrics, acute postoperative pain is commonly treated with simple analgesics that often are not very effective and frequently are used at doses lower than would be optimal (4).

Several routes of drug administration are available for pediatric patients. Oral and rectal routes are the most commonly used. Additionally, epidural and peripheral nerve blocks, wound infiltration of local anesthetics, and sublingual or transmucosal drug administration can be used for pediatric postoperative pain management. Intramuscular administration should be avoided, not only because of pain and the psychological impact but because of unpredictable drug absorption and the timing of the clinical effect (5–9).

The benefits of regional analgesia for children include safety and efficacy with a lack of increased risk when compared with general anesthesia alone. For use in children, local anesthetics should have a low risk of systemic toxicity. Motor block is a frightening experience for young children who do not understand the reason for this event, and it is therefore better to separate the sensory and motor block as much as possible (5–9).

## 2. Objectives

We compared three different methods for pediatric postoperative pain management including suppository acetaminophen, caudal block with bupivacaine, and bupivacaine wound infiltration.

## 3. Patients and Methods

This study was performed after receiving permission from the institution’s human subjects committee as well as informed consent from the participants’ parents. In this randomized, double-blinded, controlled clinical trial, 90 children between the ages of 3 months and 7 years, ASA I–II, who were scheduled for elective unilateral inguinal herniorrhaphy were assigned to three equal groups (by using colored cards). Patients with a positive history of anticonvulsive, opioid, analgesic, sedative, corticosteroid, and nonsteroidal anti-inflammatory drug consumption were excluded from this study. For premedication, 0.02 mg/kg midazolam, 1 μg/kg fentanyl, and 0.02 mg/kg atropine were administered intravenously to all patients. The patients also received 5 mg/kg sodium thiopental and 0.5 mg/kg atracurium intravenously for induction of anesthesia and isoflurane (1%) with O2 and N2O (50%–50%) during the maintenance of anesthesia.

For postoperative analgesia, one group received suppository acetaminophen (20 mg/kg). Because of the average time of operation (30–45 minutes) and the delayed onset of the action of suppository acetaminophen (30–60 minutes), the acetaminophen was administered before premedication. In the second group, bupivacaine 0.5% (2 mg/kg) was infiltrated in the wound by the surgeon, and in the third group, a single-shot caudal block with bupivacaine 0.25% (0.75 mL/kg) was performed by the anesthesia provider at the end of surgery. Because the caudal blocks in children were performed while the child was anesthetized, it was not possible to assess the effectiveness of the block by testing for sensory analgesia levels. Thus, we predicted the success of caudal block from the laxity of the anal sphincter secondary to the reduction in sphincter tone from the local anesthetic block.

Initially, 30 minutes after operation in the recovery room and then every hour during the next 6 hours of postoperative period, the FLACC pain scale was applied by one trained researcher who was not aware of the methods of analgesia.

The FLACC pain scaling system is a behavioral pain assessment scale that is used to assess pain for children between the ages of 2–7 years or individuals that are unable to communicate their pain. The scale has five criteria, and each is assigned a score of 0, 1, or 2. The FLACC is scored in a range of 0–10, with 0 representing no pain, relaxed and comfortable; 1–3: mild discomfort; 4–6: moderate pain; 7–10: severe discomfort or pain or both (10) (*Table 1*).

During the study, if the FLACC pain score was 4 or over, 0.5 mg/kg of intravenous meperidine was administered postoperatively as a supplemental analgesic and recorded.

The data were transferred to the SPSS-10 software and analyzed statistically with chi-square and analysis of variance tests. *P* < 0.05 was considered significant.

The clinical trial registration number is:

IRCT201110027695N1

## 4. Results

The study groups were comparable with respect to age, sex and weight (*Table 2*).

Significant differences between the bupivacaine infiltration group and caudal block group were not observed with regard to the duration of postoperative analgesia (FLACC < 4), but there were significant statistical differences between these two groups and the acetaminophen group (*Table 3*).

Eleven patients in the acetaminophen group (36.7%), four patients in the bupivacaine infiltration group (13.3%), and four patients in the caudal block group (13.3%) had a FLACC score ≥ 4, and they received 0.5 mg/kg intravenous meperidine for postoperative pain treatment. The mean and standard deviation of the amount of meperidine administered in the acetaminophen group, the bupivacaine infiltration group, and the caudal block group was 2.5 ± 0.97 mg, 0.66 ± 0.11 mg, and 0.83 ± 0.13 mg, respectively.

With regard to meperidine administration, significant differences were not observed between the bupivacaine infiltration and caudal block groups (*P* = 0.848), but significant statistical differences were observed between these two groups and the acetaminophen (*P* < 0.05).

**Figure 1.**
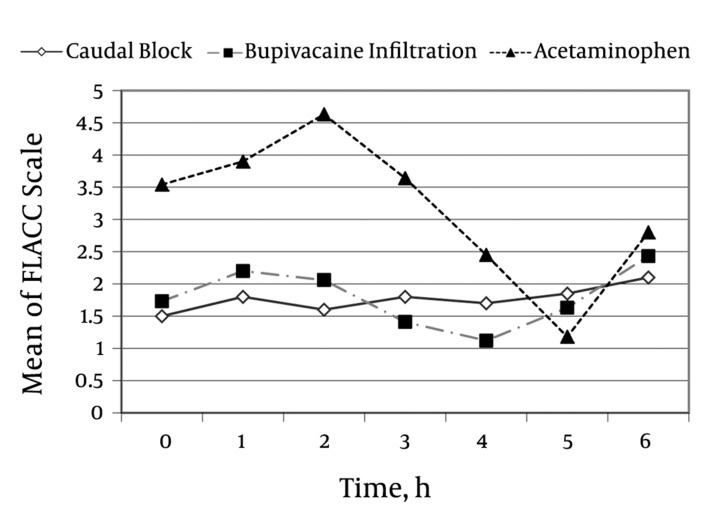
Comparison of the Mean FLACC Score in the Three Groups at Different Times

During most of the study, patients in the acetaminophen group had higher FLACC scores than those in the bupivacaine infiltration and caudal block groups. The mean FLACC score of these two groups was approximately 2 or less in the first five hours of the study. The mean FLACC score in the acetaminophen group increased significantly during the first 2 hours of the postoperative period and decreased after meperidine administration in some patients. During the last hour of the study, the FLACC score increased again in this group (*Figure 1*).

Three patients were excluded from the study because of failure of the caudal block. No major complications (hypotension, seizures, motor block, urinary retention, nausea, and vomiting) were noted in the three study groups.

**Table 1.** Criteria of the FLACC Pain Scale

Criteria	Score 0	Score 1	Score 2
Face	No particular expression or smile	Occasional grimace or frown, withdrawn, uninterested	Frequent to constant quivering chin, clenched jaw
Legs	Normal position or relaxed	Uneasy, restless, tense	Kicking, or legs drawn up
Activity	Lying quietly, normal position, moves easily	Squirming, shifting back and forth, tense	Arched, rigid or jerking
Cry	No cry (awake or asleep)	Moans or whimpers; occasional complaint	Crying steadily, screams or sobs, frequent complaints
Consolability	Content, relaxed	Reassured by occasional touching, hugging or being talked to, distracted	Difficult to console or comfort

**Table 2.** Demographic Data

	Groups			
	Acetaminophen, n = 30	Bupivacaine Infiltration, n = 30	Caudal Block, n = 30	*P* value
Age, mo, Mean ± SD	38.37 ± 21.38	37.53 ± 20.40	39.83 ± 19.76	0.701
Weight, Kg, Mean ± SD	12.03 ± 4.88	13.7 ± 4.44	13.37 ± 3.47	0.898
Sex, No.				0.355
Male	30	29	28	
Female	0	1	2	

**Table 3.** Comparison of the Duration of Analgesia in the Three Groups

	Duration of Analgesia, h	*P* value
Group I vs. Group II		0.034
Acetaminophen	4.07 ± 2.47	
Bupivacaine Infiltration	5.40 ± 1.73	
Group I vs. Group III		0.039
Acetaminophen	4.07 ± 2.47	
Caudal Block	5.37 ± 1.79	
Group II vs. Group III		0.9
Bupivacaine Infiltration	5.40 ± 1.73	
Caudal Block	5.37 ± 1.79	

## 5. Discussion

Pain is a subjective symptom that can be difficult to evaluate with regard to intensity, duration, tolerance, and threshold in pediatrics. Postoperative pain is a subjective symptom that has been extensively studied in adults but only minimally in children. The use of low analgesic doses and failure to document the pain and its management are common concerns in pediatric patients (4).

There is an incorrect assumption that young children do not have well-developed pain pathways and therefore do not require as much analgesia as older patients. In young children who are unable to express pain verbally, crying is sometimes attributed to the mothers’ absence, hunger, or the unfamiliar hospital environment rather than pain (4, 11, 12). Pain in children causes distress not only for them but also for their parents and the medical staff. Pain in newborns, infants, and children has the same negative effects as in adults (5–9).

Thus, it is now widely accepted that infants and children require appropriate pain relief in the post-operative period. Postoperative pain management not only minimizes patient suffering but also can reduce morbidity and facilitate rapid recovery and early discharge from hospital, which can reduce hospital costs (2).

Some studies have evaluated which analgesic technique is ideal to use during the postoperative period for pediatric surgeries. Wheeler *et al.* demonstrated that the addition of clonidine did not enhance the postoperative analgesia of a caudal block using 0.125% bupivacaine and epinephrine and did not significantly decrease the overall need for rescue analgesics in children aged 2–8 years and undergoing surgical procedures below the umbilicus (13).

In another study in Australia, caudal analgesia, landmark-based penile block and penile block under ultrasonography were compared for circumcision in children, and it was demonstrated that caudal block and penile block under ultrasonography resulted in a longer duration of postoperative analgesia (14).

In the other study, the postoperative analgesic effect of suppository paracetamol was compared with the combination of suppository paracetamol and bupivacaine wound infiltration for inguinal herniorrhaphy. The combination of these two methods produced better analgesia than suppository paracetamol alone (15).

Razavi and colleagues compared suppository acetaminophen and caudal anesthesia in relieving pain after pediatric surgery and concluded that caudal block was more effective than suppository acetaminophen (16).

The purpose of our study was to compare the effects of suppository acetaminophen, bupivacaine wound infiltration, and caudal block with bupivacaine on postoperative pain in pediatric inguinal herniorrhaphy. In this study, there was no significant statistical difference among the three groups with regard to age, sex, and weight. From this study, we can infer that bupivacaine wound infiltration and caudal block with bupivacaine provide a similar postoperative analgesic effect. They are also more effective than suppository acetaminophen for controlling pediatric postoperative pain.

In conclusion, the performance of caudal block requires experience and may lead to complications, and block failure is not unusual. In contrast, wound infiltration is simple without significant side effects or failure. Thus, bupivacaine wound infiltration may be a preferred technique for producing postoperative analgesia in pediatric inguinal herniorrhaphy.
